# Caveolin-1 levels associated with coronary artery disease in Chinese participants: a retrospective cohort study

**DOI:** 10.3389/fcvm.2026.1790930

**Published:** 2026-08-07

**Authors:** Jiazheng Chen, Hongli Li, Yuxin Liang, Xiaoyang Wang, Zhijun Meng

**Affiliations:** 1 School of the Fifth Clinical Medicine, Shanxi Medical University, Taiyuan, China; 2 Department of Clinical Laboratory Diagnostics, Shanxi Provincial People’s Hospital, Shanxi Medical University, Taiyuan, China; 3 Department of Medical Technology, Shanxi Provincial People’s Hospital, Shanxi Medical University, Taiyuan, China; 4 Deparment of Clinical Laboratory, Shanxi Provincial People’s Hospital, Taiyuan, China

**Keywords:** albumin, biomarker, caveolin-1, Chinese population, coronary artery disease

## Abstract

**Background:**

Coronary artery disease (CAD) remains a leading cause of mortality globally, yet approximately 30% of CAD risk remains unexplained by conventional factors. Caveolin-1 (Cav-1) regulates endothelial function through inhibiting endothelial nitric oxide synthase and promoting LDL transcytosis. While genome-wide association studies linked CAV1 variants to CAD risk (OR 1.19–1.23), whether circulating Cav-1 serves as a clinical biomarker for CAD risk stratification remains unknown.

**Methods:**

This retrospective cohort study enrolled 160 participants (105 CAD patients, 55 controls) who underwent coronary angiography at Shanxi Provincial People's Hospital, China, from September 2024 to February 2025. CAD was defined as ≥50% stenosis in major coronary arteries. Serum Cav-1 levels were measured using ELISA. Multivariable logistic regression models adjusted for age, sex, lipids, and biomarkers evaluated Cav-1's independent association with CAD. Restricted cubic spline (RCS) analysis assessed dose-response relationships, and receiver operating characteristic (ROC) curves evaluated discriminative performance.

**Results:**

CAD patients exhibited significantly lower Cav-1 levels than controls (7.0 ± 2.7 vs. 8.5 ± 2.7 ng/mL, *P* = 0.001). After full adjustment, each 1 ng/mL increase in Cav-1 conferred 18% CAD risk reduction (OR 0.82, 95% CI 0.68–0.98, *P* = 0.031). Participants in the highest Cav-1 quartile demonstrated 80% lower risk vs. the lowest quartile (OR 0.20, 95% CI 0.05–0.84, *P* = 0.028; *P* for trend <0.01). RCS analysis revealed a consistent linear inverse relationship (*P* for nonlinearity = 0.812). Combining Cav-1 with albumin significantly enhanced discrimination (AUC 88.4%, 95% CI 83.3–93.5), approaching the full multivariable model performance (AUC 89.8%).

**Conclusions:**

Circulating Cav-1 demonstrates an independent, linear inverse association with CAD risk in Chinese adults. Combined with albumin, Cav-1 achieves robust discriminative performance, suggesting potential utility for refined risk stratification beyond traditional risk factors.

## Introduction

Coronary artery disease (CAD) remains a leading cause of global morbidity and mortality, accounting for over 9 million deaths globally each year (1). In China, coronary artery disease (CAD) has become an increasingly significant health challenge. Recent surveillance data (2020–2022) showed that the prevalence of CAD among adults aged ≥18 years was 758/100,000, with approximately 11.39 million patients nationwide. Moreover, CAD mortality has been rising steadily since 2012, reaching over 140/100,000 by 2021 (2). Although traditional and emerging risk factors collectively account for approximately 70% of CAD risk, the PURE study revealed that metabolic factors contributed 41.2% of the population attributable fraction (PAF) for CVD, with hypertension alone accounting for 22.3%. This indicates that nearly 30% of CAD risk remains unexplained by conventional risk assessment (3), underscoring the need for novel biomarkers to refine risk stratification.

Caveolin-1 (Cav-1), a structural protein of caveolae, regulates endothelial function and lipid metabolism (4). Preclinical studies demonstrate Cav-1's dual and independent roles in cardiovascular disease. Cav-1 tonically inhibits endothelial nitric oxide synthase (eNOS) through direct binding to the enzyme via its scaffolding domain (amino acids 82–101), particularly the F92 residue (5). Besides, Cav-1 promotes atherosclerosis via LDL transcytosis, with Cav-1 deficiency conferring atheroprotection by inhibiting endothelial LDL transport and reducing pro-inflammatory fibronectin deposition (6). Genome-wide association studies validated that CAV1 variants increasing expression confer higher risk for CAD/myocardial infarction (OR = 1.19–1.23) in Chinese populations (7) and metabolic syndrome (OR = 1.61–2.83) in Caucasian/ Hispanic cohorts (8).

Significant knowledge gaps remain regarding the clinical application value of Cav-1 in coronary artery disease (CAD). First, no studies have yet evaluated the role of circulating Cav-1 as a biomarker for CAD risk stratification, and whether serum Cav-1 measurements can provide incremental predictive value beyond established risk factors (LDL-C, HDL-C, inflammatory markers) remains unknown (9). Second, although genetic studies have established an association between CAV1 and CAD (7), the quantitative relationship between circulating Cav-1 expression levels and atherosclerotic burden has not been explored in clinical cohorts.

In this study, we hypothesize that serum Cav-1 level is associated with CAD risk in Chinese adults and synergizes with metabolic biomarkers to enhance prediction. This retrospective cohort study aims to: (1) quantify Cav-1's adjusted association with CAD, (2) model its dose-response curve using spline regression, and (3) evaluate its discriminative utility alone and combined with routine biomarkers.

## Methods

### Study design and sample

This study is a retrospective observational study conducted at Shanxi Provincial People's Hospital in Taiyuan, China in September 2024. The research protocol received formal approval from the Ethics Committee of Shanxi Provincial People's Hospital [Ethics Approval Number: (2023) Provincial Medical Ethics Review Document No. 108]. The study strictly adhered to the principles stipulated in the Declaration of Helsinki and followed Good Clinical Practice (GCP) guidelines.

The study enrolled 160 adult participants in two groups. The CAD group (*n* = 105) consisted of patients aged 30–90 years who underwent coronary angiography due to suspected coronary artery disease and were diagnosed with CAD (defined as ≥50% stenosis in at least one major coronary artery). The control group (*n* = 55) consisted of individuals without clinical signs or diagnostic evidence of coronary artery disease, recruited contemporaneously from the health examination center of the same hospital.

### Inclusion and exclusion criteria

Inclusion criteria for the CAD group were: (1) age ≥18 years; (2) complete clinical data and laboratory results available; (3) CAD confirmed by coronary angiography. Inclusion criteria for the control group were: (1) age ≥18 years; (2) complete clinical data and laboratory results available; (3) no prior history or clinical evidence of coronary artery disease.

Exclusion criteria for both groups included: (1) incomplete medical records or missing key laboratory parameters; (2) known malignancy or severe systemic disease; (3) acute infectious disease within the past month; (4) chronic hepatic or renal failure; (5) history of previous coronary artery bypass grafting or percutaneous coronary intervention.

Demographic characteristics, clinical parameters, and laboratory test results were systematically collected from the hospital electronic medical record system.

### Laboratory measurements

Fasting venous blood samples (10 mL) were collected from the antecubital vein of each participant after an overnight fast (≥8 h). Samples were centrifuged at 3,000 rpm for 10 min at 4 °C, and serum/plasma aliquots were carefully transferred into Eppendorf tubes and stored at −80 °C until analysis. All samples underwent only one freeze-thaw cycle before laboratory testing. Serum Cav-1 concentrations were measured using a commercially available human Cav-1 enzyme-linked immunosorbent assay (ELISA) kit [catalogue number: (NBP2-75052), Novus Biologicals, St. Louis, Missouri, USA] according to the manufacturer's instructions. The kit had a detection range of 0.16–10 ng/mL, a sensitivity of 0.10 ng/mL, and a minimum detectable concentration of 0.31 ng/mL. Spike recovery was 85%–102%, with intra- and inter-assay CVs of <5.16% and <4.88%, respectively. Dilution linearity was confirmed, and no matrix interference or cross-reactivity was observed. Other laboratory parameters were measured using standardised automated analyser systems in the Department of Clinical Laboratory, Shanxi Provincial People's Hospital. Haematological parameters (white blood cell count, haemoglobin, platelet count, packed cell volume, and) were analysed using an automated haematology analyser (Sysmex, Kobe, Japan). Biochemical parameters [serum creatinine, blood urea nitrogen (BUN), albumin, potassium, sodium, and chloride] and metabolic markers (glycated, fasting glucose, homocysteine, total cholesterol (TC), high-density lipoprotein cholesterol (HDL-C), low-density lipoprotein cholesterol (LDL-C), and triglycerides (TG)) were measured on an automated biochemistry analyser (Beckman Coulter, Brea, USA). using standard laboratory techniques. Cardiovascular biomarkers, including high-sensitivity cardiac troponin (hs-cTn), and B-type natriuretic peptide (BNP), were quantified using chemiluminescent immunoassays (Beckman Coulter, Brea USA). All measurements were performed by experienced laboratory technicians blinded to participants' clinical status. In addition, prothrombin time was measured using a fully automated coagulation analyzer (Werfen, Barcelona, Spain), whereas glycated hemoglobin (HbA1c) was analyzed on a dedicated, fully automated glycated hemoglobin analyzer (Bio-Rad, California, USA). The cholesterol-glucose index (CHG) is calculated using the formula: log[TC [mmol/L] × glucose [mmol/L]/HDL [mmol/L]/2], where TC denotes total cholesterol and HDL denotes high-density lipoprotein cholesterol.

### Statistical analysis

Baseline characteristics were summarized for the entire cohort (*n* = 160) and analyzed in two ways: by caveolin-1 quartiles (Q1–Q4, *n* = 40 each) and by coronary artery disease status (No CAD, *n* = 55; CAD, *n* = 105). For normally distributed continuous variables, data are presented as mean ± standard deviation and were compared using one-way ANOVA across quartiles and independent-samples *t*-test between CAD groups. Non-normally distributed continuous variables are reported as median (interquartile range) and were analyzed using the Kruskal–Wallis test for quartile comparisons and the Mann–Whitney *U*-test for CAD status comparisons. Categorical variables are presented as counts and percentages and were compared using chi-square test or Fisher's exact test when expected cell counts were small. For all analyses, two-tailed *P* values <0.05 were considered statistically significant.

Multivariable logistic regression models evaluated the independent association between Cav-1 levels and Coronary Artery Disease (CAD). Three analytical tiers were implemented: Model 1 (unadjusted), Model 2 (adjusted for age and sex), and Model 3 (adjusted for age, sex, TC, HDL-C, BNP, and BUN). Covariates for Model 3 were selected via a standardized protocol: (1) significant univariate association with CAD (*P* < 0.05), (2) ≥ 10% change in the Cav-1 effect estimate upon inclusion/exclusion, and (3) variance inflation factor (VIF)<2.0 to exclude multicollinearity. Results are reported as odds ratios (ORs) with 95% confidence intervals (CIs). Statistical significance was defined as two-tailed *P* < 0.05. The P for trend was derived by assigning median values to quartiles as continuous variables.

To evaluate the potential non-linear relationship between Cav-1 levels and Coronary Artery Disease (CAD), we employed restricted cubic spline logistic regression analyses with knots placed at the 10th, 50th, and 90th percentiles. The reference point was set at 7.134 ng/mL (median value). Three models were constructed: an unadjusted model; Model 2 adjusted for demographic and hematological factors (age, sex, HbA1c, WBC, and platelet count); and Model 3 adjusted for metabolic parameters (fasting glucose, TC, HDL, LDL, and homocysteine). Both overall association and non-linearity were assessed, with *P* < 0.05 considered statistically significant.

To evaluate the incremental predictive value of Cav-1 beyond albumin, we compared a reference model (age + albumin) with an extended model (age + albumin + Cav-1). Discrimination improvement was assessed using DeLong's test for the difference in area under the receiver operating characteristic curve (ΔAUC). Reclassification improvement was quantified using categorical and continuous net reclassification improvement (NRI) and integrated discrimination improvement (IDI), as described by Pencina et al. (10). The 95% confidence intervals for NRI and IDI were estimated by bootstrapping with 500 resamples. A two-sided *p*-value <0.05 was considered statistically significant.

Discriminative performance for prevalent coronary artery disease was assessed using receiver operating characteristic (ROC) analysis. Area under the ROC curve (AUC) with 95% confidence intervals (CIs) was calculated for caveolin-1 and individual clinical/biochemical variables. Incremental discriminatory value was evaluated by estimating AUCs for models combining caveolin-1 with each covariate and for a full multivariable model. AUCs and CIs were obtained nonparametrically; two-sided *P* < 0.05 was considered indicative of statistical significance for planned comparisons.

Subgroup analyses were conducted by stratifying participants according to sex, body mass index (BMI < 24 vs. ≥ 24 kg/m^2^, based on Chinese obesity criteria), diabetes mellitus status, and hypertension status. Within each stratum, the association between Caveolin-1 and CAD was assessed using logistic regression adjusted for age and sex (except that sex was excluded from adjustment in sex-stratified analyses). Effect modification was evaluated by incorporating a multiplicative interaction term (Caveolin-1 × subgroup variable) into the logistic regression model, and the corresponding P for interaction was reported.

All analyses were performed using the statistical software R (http://www.R-project.org, The R Foundation) and Free Statistics V2.3. A two-tailed *P* value of less than 0.05 was considered statistically significant.

## Results

### Comparison of baseline characteristics between CAD and Non-CAD groups

As delineated in Table 1, participants with CAD were significantly older (62.5 ± 11.1 vs. 53.5 ± 13.4 years, *P* < 0.001) and had a lower proportion of males (66.7% vs. 96.4%, *P* < 0.001) compared to the non-CAD group. The CAD cohort also exhibited a higher BMI (25.0 ± 3.1 vs. 23.4 ± 3.0 kg/m^2^, *P* = 0.003) and greater prevalence of hypertension (59.0% vs. 27.3%, *P* < 0.001) and diabetes mellitus (31.4% vs. 12.7%, *P* = 0.009). Notably, statin use was markedly more prevalent in the CAD group (86.3% vs. 25.5%, *P* < 0.001), as was ACEI use (79.0% vs. 5.5%, *P* < 0.001), reflecting the established pharmacological management of CAD in this cohort. Smoking prevalence did not differ significantly between groups (47.1% vs. 38.2%, *P* = 0.285).

**Table 1:** Baseline characteristics of participants according to coronary artery disease status.

Characteristics	Total (*n* = 160)	Non-CAD (*n* = 55)	CAD (*n* = 105)	*P* value
Demographics				
Male, *n* (%)	123 (76.9)	53 (96.4)	70 (66.7)	<0.001*
Age, years	59.3 ± 12.7	53.5 ± 13.4	62.5 ± 11.1	<0.001*
Clinical characteristics				
Smoking, *n* (%)	69 (43.9)	21 (38.2)	48 (47.1)	0.285
Hypertension, *n* (%)	77 (48.1)	15 (27.3)	62 (59.0)	<0.001*
Diabetes mellitus, *n* (%)	40 (25.0)	7 (12.7)	33 (31.4)	0.009*
Statin use, *n* (%)	102 (65.0)	14 (25.5)	88 (86.3)	<0.001*
ACEI use, *n* (%)	86 (53.8)	3 (5.5)	83 (79.0)	<0.001*
Haematological parameters				
WBC, ×10⁹/L	6.2 ± 1.7	6.0 ± 1.5	6.4 ± 1.8	0.209
Haemoglobin, g/L	140.4 ± 15.5	140.1 ± 13.3	140.6 ± 16.6	0.858
Platelet, ×10⁹/L	219.5 ± 67.8	232.9 ± 61.7	212.4 ± 70.0	0.069
PCV	0.40 ± 0.04	0.40 ± 0.04	0.40 ± 0.05	0.551
Prothrombin time, s	11.2 ± 1.6	11.1 ± 1.0	11.3 ± 1.8	0.583
Metabolic markers				
HbA1c, %	6.1 ± 0.9	5.6 ± 0.7	6.3 ± 0.9	0.001*
Fasting glucose, mmol/L	5.2 ± 1.4	5.4 ± 1.3	5.1 ± 1.5	0.275
Total cholesterol, mmol/L	3.8 ± 1.1	4.1 ± 0.8	3.6 ± 1.3	0.010*
HDL-C, mmol/L	1.2 ± 0.3	1.3 ± 0.2	1.1 ± 0.3	<0.001*
LDL-C, mmol/L	2.3 ± 0.8	2.6 ± 0.6	2.2 ± 0.9	0.008*
CHG	2.1 ± 0.4	2.1 ± 0.2	2.1 ± 0.4	0.858
Biochemical parameters				
Serum creatinine, μmol/L	67.2 (58.2–74.8)	66.3 (59.8–74.6)	67.4 (57.3–74.9)	0.600
BUN, mmol/L	5.4 (4.4–6.3)	4.8 (4.1–5.8)	5.6 (4.7–7.0)	<0.001*
Albumin, g/L	40.9 ± 3.9	43.8 ± 2.7	39.3 ± 3.5	<0.001*
Potassium, mmol/L	3.9 ± 0.4	3.8 ± 0.3	3.9 ± 0.4	0.298
Sodium, mmol/L	139.7 ± 3.3	139.6 ± 3.5	139.8 ± 3.1	0.652
Chloride, mmol/L	106.3 ± 2.4	105.9 ± 2.3	106.5 ± 2.5	0.135
Cardiovascular biomarkers				
Homocysteine, μmol/L	16.6 ± 9.3	13.2 ± 5.3	17.8 ± 10.1	0.014*
hs-cTn, ng/L	4.3 (2.5–8.2)	5.8 (3.3–7.5)	3.7 (2.2–11.6)	0.209
BNP, pg/mL	42.0 (21.8–68.0)	36.0 (23.0–52.2)	43.0 (18.6–98.0)	0.071
Caveolin-1 level	7.5 ± 2.8	8.5 ± 2.7	7.0 ± 2.7	0.001*

Data are presented as mean ± SD for normally distributed continuous variables, median (IQR) for non-normally distributed continuous variables, or *n* (%) for categorical variables. Non-normally distributed variables analysed using Mann–Whitney *U*-test; all other continuous variables analysed using independent samples *t*-test or ANOVA; categorical variables analysed using *χ*^2^-test.

* Statistically significant at *P* < 0.05.

BNP, B-type natriuretic peptide; BUN, blood urea nitrogen; CAD, Coronary Artery Disease; CVA-1, cerebrovascular accident-1; HbA1c, glycated haemoglobin; HDL-C, high-density lipoprotein cholesterol; hs-cTn, high-sensitivity cardiac troponin; IQR, interquartile range; LDL-C, low-density lipoprotein cholesterol; PCV, packed cell volume; SD, standard deviation; WBC, white blood cell count.

In terms of metabolic and biochemical parameters, the CAD cohort exhibited elevated HbA1c (6.3 ± 0.9% vs. 5.6 ± 0.7%, *P* = 0.01), homocysteine (17.8 ± 10.1 vs. 13.2 ± 5.3 μmol/L, *P* = 0.014), and BUN (median 5.6 vs. 4.8 mmol/L, *P* < 0.001), alongside reduced albumin (39.3 ± 3.5 vs. 43.8 ± 2.7 g/L, *P* < 0.001), total cholesterol (3.6 ± 1.3 vs. 4.1 ± 0.8 mmol/L, *P* = 0.010), HDL-C (1.1 ± 0.3 vs. 1.3 ± 0.2 mmol/L, *P* < 0.001), LDL-C (2.2 ± 0.9 vs. 2.6 ± 0.6 mmol/L, *P* = 0.008), and caveolin-1 levels (7.0 ± 2.7 vs. 8.5 ± 2.7 ng/mL, *P* = 0.001). The paradoxically lower LDL-C in the CAD group is most plausibly attributable to the substantially higher prevalence of statin use in this group. No significant differences were observed in haematological parameters, electrolytes, CHG, or cardiovascular biomarkers (hs-cTn, BNP) (*P* > 0.05). These results delineate distinct clinical, metabolic, and biomarker profiles associated with CAD status.

### Baseline characteristics stratified by Cav-1 Levels

As detailed in Table 2, Coronary Artery Disease (CAD) prevalence decreased progressively across ascending caveolin-1 quartiles (Q1: 80.0% vs. Q4: 45.0%, *P* < 0.001). Participants in higher quartiles were younger (Q1: 62.6 ± 13.6 vs. Q4: 54.7 ± 11.8 years, *P* = 0.028). Regarding clinical characteristics, ACEI use differed significantly across quartiles (Q1: 65.0% vs. Q4: 40.0%, *P* = 0.017), with higher use observed in lower Cav-1 quartiles, consistent with the greater CAD burden in these groups. Statin use, hypertension, diabetes mellitus, and smoking prevalence showed no statistically significant differences across quartiles (*P* = 0.056, 0.058, 0.083, and 0.923, respectively), though a trend toward higher statin use in Q1–Q2 was noted (70.0% and 79.5% vs. 57.5% and 52.6% in Q3–Q4). Hemoglobin levels varied significantly (*P* = 0.023), with the highest value observed in Q3 (146.6 ± 15.7 g/L). Lipid profiles demonstrated positive associations with caveolin-1 levels: total cholesterol (Q1: 3.3 ± 0.8 vs. Q4: 4.2 ± 1.4 mmol/L, *P* = 0.002), HDL-C (Q1: 1.1 ± 0.3 vs. Q4: 1.3 ± 0.3 mmol/L, *P* = 0.031), and LDL-C (Q1: 2.0 ± 0.6 vs. Q4: 2.6 ± 1.0 mmol/L, *P* < 0.001). Albumin differed across quartiles (*P* = 0.022), peaking in Q3 (42.2 ± 4.2 g/L), while hematocrit (HCT) varied significantly (*P* = 0.014). No significant differences were observed for sex distribution, hematologic parameters (WBC, platelet), metabolic markers (HbA1c, glucose), electrolytes, or cardiovascular biomarkers (homocysteine, hs-cTn, BNP). These findings demonstrate distinct demographic, clinical, and metabolic patterns across caveolin-1 quartiles.

**Table 2:** Baseline characteristics and coronary artery disease prevalence according to caveolin-1 quartiles.

Characteristics	Total (*n* = 160)	Q1 (*n* = 40)	Q2 (*n* = 40)	Q3 (*n* = 40)	Q4 (*n* = 40)	*P* value
Primary outcome						
CAD, *n* (%)	105 (65.6)	32 (80.0)	33 (82.5)	22 (55.0)	18 (45.0)	<0.001*
Demographics						
Male, *n* (%)	123 (76.9)	31 (77.5)	27 (67.5)	34 (85.0)	31 (77.5)	0.323
Age, years	59.3 ± 12.7	62.6 ± 13.6	61.5 ± 10.2	58.7 ± 13.8	54.7 ± 11.8	0.028*
Clinical characteristics						
Smoking, *n* (%)	69 (43.9)	19 (48.7)	16 (42.1)	17 (42.5)	17 (42.5)	0.923
Hypertension, *n* (%)	77 (48.1)	26 (65.0)	20 (50.0)	16 (40.0)	15 (37.5)	0.058
Diabetes mellitus, *n* (%)	40 (25.0)	15 (37.5)	10 (25.0)	10 (25.0)	5 (12.5)	0.083
Statin use, *n* (%)	102 (65.0)	28 (70.0)	31 (79.5)	23 (57.5)	20 (52.6)	0.056
ACEI use, *n* (%)	86 (53.8)	26 (65.0)	27 (67.5)	17 (42.5)	16 (40.0)	0.017*
Haematological parameters						
WBC, ×10⁹/L	6.2 ± 1.7	6.3 ± 1.9	6.3 ± 2.0	6.3 ± 1.4	6.1 ± 1.5	0.906
Haemoglobin, g/L	140.4 ± 15.5	140.1 ± 16.4	137.2 ± 14.1	146.6 ± 15.7	137.7 ± 14.3	0.023*
Platelet, ×10⁹/L	219.5 ± 67.8	215.1 ± 63.2	212.0 ± 55.7	219.7 ± 90.8	231.0 ± 56.2	0.616
PCV	0.40 ± 0.04	0.40 ± 0.04	0.40 ± 0.04	0.43 ± 0.04	0.40 ± 0.04	0.014*
Prothrombin time, s	11.2 ± 1.6	11.7 ± 2.7	11.2 ± 1.0	11.1 ± 0.8	11.0 ± 1.0	0.254
Metabolic markers						
HbA1c, %	6.1 ± 0.9	6.4 ± 1.1	6.0 ± 0.7	6.0 ± 1.0	5.8 ± 0.8	0.113
Fasting glucose, mmol/L	5.2 ± 1.4	5.5 ± 1.5	4.9 ± 1.1	5.5 ± 1.8	5.0 ± 0.9	0.144
Total cholesterol, mmol/L	3.8 ± 1.1	3.3 ± 0.8	3.7 ± 1.2	4.1 ± 0.9	4.2 ± 1.4	0.002*
HDL-C, mmol/L	1.2 ± 0.3	1.1 ± 0.3	1.1 ± 0.3	1.2 ± 0.2	1.3 ± 0.3	0.031*
LDL-C, mmol/L	2.3 ± 0.8	2.0 ± 0.6	2.2 ± 0.7	2.5 ± 0.7	2.6 ± 1.0	<0.001*
Biochemical parameters						
Serum creatinine, μmol/L	67.2 (58.2–74.8)	70.3 (60.5–76.1)	66.3 (56.1–76.3)	68.8 (61.0–74.0)	60.8 (56.9–70.1)	0.074
BUN, mmol/L	5.4 (4.4–6.3)	5.8 (5.1–7.0)	5.3 (4.3–6.3)	5.1 (4.3–5.8)	5.3 (4.1–6.0)	0.097
Albumin, g/L	40.9 ± 3.9	40.3 ± 3.9	39.6 ± 3.1	42.2 ± 4.2	41.5 ± 3.9	0.022*
Potassium, mmol/L	3.9 ± 0.4	3.9 ± 0.3	3.8 ± 0.4	3.8 ± 0.4	3.9 ± 0.4	0.706
Sodium, mmol/L	139.7 ± 3.3	139.3 ± 3.1	140.1 ± 2.9	139.9 ± 3.3	139.6 ± 3.7	0.777
Chloride, mmol/L	106.3 ± 2.4	106.4 ± 2.4	107.1 ± 2.4	105.7 ± 2.5	106.0 ± 2.1	0.077
Cardiovascular biomarkers						
Homocysteine, μmol/L	16.6 ± 9.3	16.5 ± 8.1	15.9 ± 8.7	17.4 ± 10.8	16.6 ± 10.2	0.941
hs-cTn, ng/L	4.3 (2.5–8.2)	3.7 (3.1–7.7)	3.7 (2.3–8.6)	4.4 (2.3–7.5)	5.8 (3.5–8.2)	0.466
BNP, pg/mL	42.0 (21.8–68.0)	30.1 (15.0–75.0)	48.0 (23.0–78.0)	30.5 (20.2–64.2)	43.0 (21.5–67.2)	0.534

Data are presented as mean ± SD for normally distributed continuous variables, median (IQR) for non-normally distributed continuous variables, or *n* (%) for categorical variables. Q1 represe*n*ts the lowest quartile and Q4 the highest quartile of Caveolin-1 levels. Non-normally distributed variables analysed using Kruskal–Wallis test; continuous variables analysed using one-way ANOVA; categorical variables analysed using *χ*^2^-test. P for trend was calculated using linear-by-linear association test for categorical variables and linear regression for continuous variables.

* Statistically significant at *P* < 0.05.

BNP, B-type natriuretic peptide; BUN, blood urea nitrogen; CAD, Coronary Artery Disease; HbA1c, glycated haemoglobin; HDL-C, high-density lipoprotein cholesterol; hs-cTn, high-sensitivity cardiac troponin; IQR, interquartile range; LDL-C, low-density lipoprotein cholesterol; PCV, packed cell volume; Q, quartile; SD, standard deviation; WBC, white blood cell count.

**Quartile definition:** Participants were divided into quartiles based on Caveolin*-*1 levels: Q1 (lowest, ≤5.58 ng/mL), Q2 (5.59–7.13 ng/mL), Q3 (7.14–8.93 ng/mL), and Q4 (highest, >8.93 ng/mL).

### Multivariable-Adjusted association of Cav-1 with CAD

As detailed in Table 3, continuous Cav-1 levels demonstrated a consistent inverse association with CAD risk across all models (Model 1: OR 0.82, 95% CI 0.72–0.93, *P* = 0.002; Model 3: OR 0.82, 0.68–0.98, *P* = 0.031). Quartile-based analysis revealed a significant dose-response relationship: participants in Q4 exhibited markedly lower CAD risk vs. Q1 (Model 1: OR 0.20, 95% CI 0.08–0.55, *P* = 0.002; Model 3: OR 0.20, 0.05–0.84, *P* = 0.028). A statistically significant inverse trend persisted across quartiles (*P* for trend ≤0.01 in all models). Q3 showed a trend toward reduced risk (Model 3: OR 0.28, 0.07–1.09, *P* = 0.067), while Q2 demonstrated no significant association. These results confirm a robust, independent protective effect of higher Cav-1 levels against CAD after rigorous adjustment for cardiovascular risk factors.

**Table 3:** Association between caveolin-1 levels and coronary artery disease: multivariable logistic regression analysis.

**Exposure**	**Model1**		**Model2**		**Model3**	
	**OR (95%CI)**	***P* value**	**OR (95%CI)**	***P* value**	**OR (95%CI)**	***P* value**
**Caveolin-1**	0.82 (0.72∼0.93)	0.002	0.82 (0.71∼0.94)	0.006	0.82 (0.68∼0.98)	0.031
**Caveolin-1 quintiles**						
Q1	1(Ref)		1(Ref)		1(Ref)	
Q2	1.18 (0.38∼3.63)	0.775	1.15 (0.35∼3.78)	0.816	0.86 (0.19∼3.86)	0.841
Q3	0.31 (0.11∼0.83)	0.019	0.36 (0.12∼1.05)	0.06	0.28 (0.07∼1.09)	0.067
Q4	0.2 (0.08∼0.55)	0.002	0.21 (0.07∼0.65)	0.006	0.2 (0.05∼0.84)	0.028
*P* for trend	0.53 (0.39∼0.74)	<0.001	0.55 (0.39∼0.79)	0.001	0.55 (0.35∼0.87)	0.01

**Mod*e*l** **1:** Crude model without covariate adjustment.

***M*odel 2:** Adjusted for age and sex.

***M*odel 3:** Adjusted for age, sex, total cholesterol, high-density lipoprotein cholesterol, B-type natriuretic peptide, and blood urea nitrogen. Covariates were selected based on a standardised protocol: (1) statistically significant association with CAD in univariable analysis (*P* < 0.05); (2) change in the effect estimate (OR) for Caveolin-1 of ≥10% when included in or excluded from the model, indicating confounding; and (3) variance inflation factor <2.0 to exclude multicollinearity.

*P* for trend was calculated by entering the median value of each quartile as a continuous variable in the logistic regression model.

CAD, Coronary Artery Disease; CI, confidence interval; OR, odds ratio; Q, quartile; Ref, reference category.

*C*aveolin-1 quartile cut-off values (ng/mL): Q1 ≤ 5.58, Q2 5.59–7.13, Q3 7.14–8.93, Q4 > 8.93 (range 0.64–16.00).

### Restricted cubic spline analysis of Cav-1 and CAD risk

**Figure 1:**
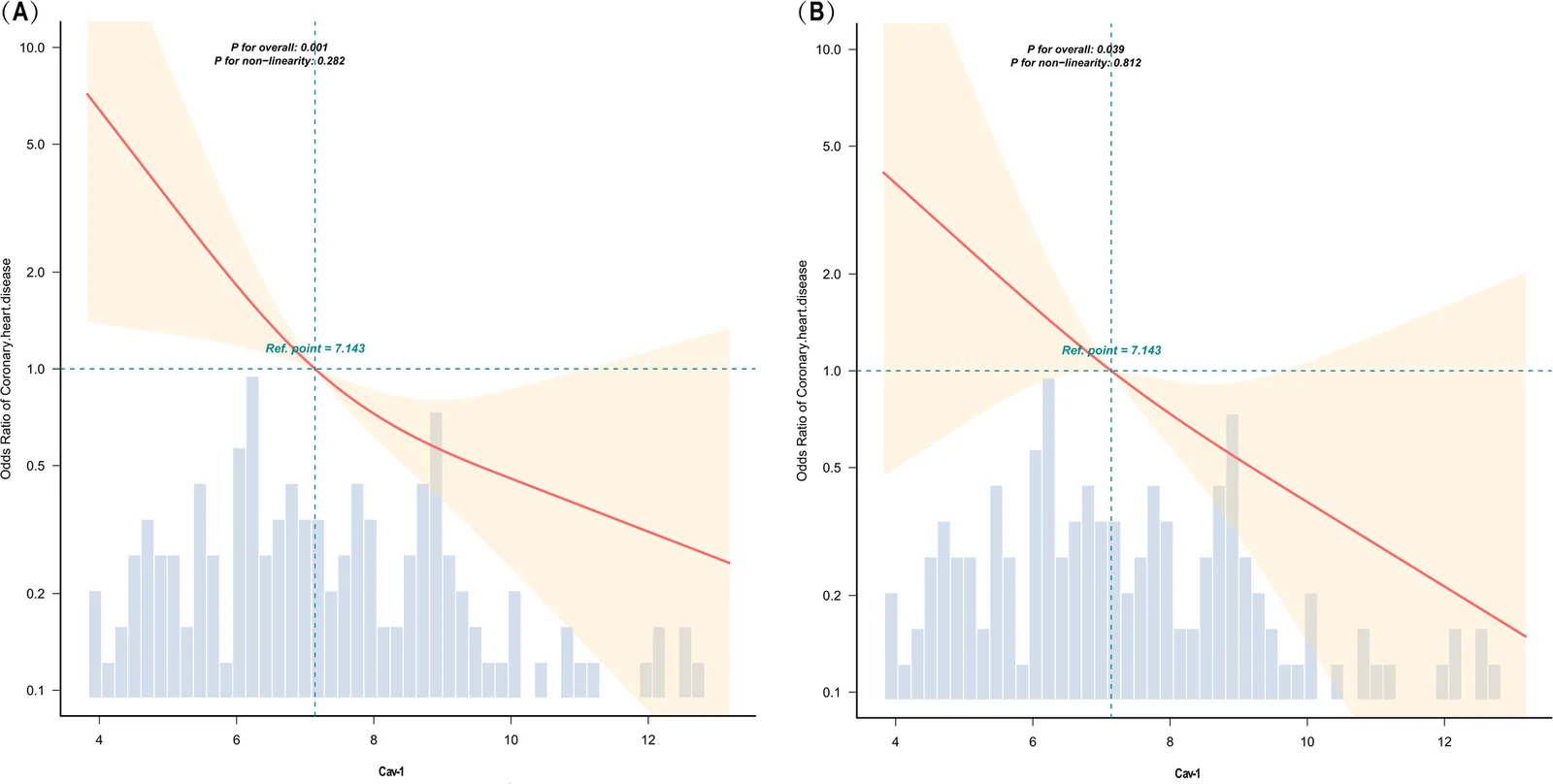
Dose-response relationship between caveolin-1 levels and coronary heart disease. Restricted cubic spline curves with 3 knots (positioned at the 10th, 50th, and 90th percentiles) illustrating the association between Caveolin-1 levels and odds ratios (ORs) for coronary heart disease. The median Caveolin-1 value (7.143 units) serves as the reference point (OR = 1.0, indicated by the horizontal dashed grey line and vertical dashed blue line): association without covariate adjustment. The red solid line represents the estimated OR, and the shaded orange area denotes the 95% confidence interval (CI). **(A)** Model 1 (unadjusted model): Crude association without covariate adjustment. **(B)** Model 3 (fully adjusted model): Adjusted for age, sex, total cholesterol, high-density lipoprotein cholesterol, B-type natriuretic peptide, and blood urea nitrogen.

Figure 1 displays the dose-response curves between Cav-1 and coronary heart disease risk. In the unadjusted model (Panel A), a significant inverse association was observed (*P* < 0.001), with odds ratios declining from 7.5 at Caveolin-1 = 4 units to 0.2 at 13 units. The relationship was essentially linear (P for non-linearity = 0.292). After adjusting for cardiovascular risk factors (Panel B), the inverse association persisted (*P* = 0.019) with comparable linearity (*P* for non-linearity = 0.812). At Cav-1 levels of 5 units, the adjusted OR was 3.5 (95% CI 1.2–10.5), decreasing to 0.3 (95% CI 0.1–0.9) at 12 units. The absence of significant non-linearity in both models supports a consistent linear inverse relationship between Cav-1 and coronary heart disease risk across the observed concentration range, indicating that higher Cav-1 levels confer progressive cardioprotection.

### ROC curve analysis for CAD prediction using Cav-1-based models

**Figure 2:**
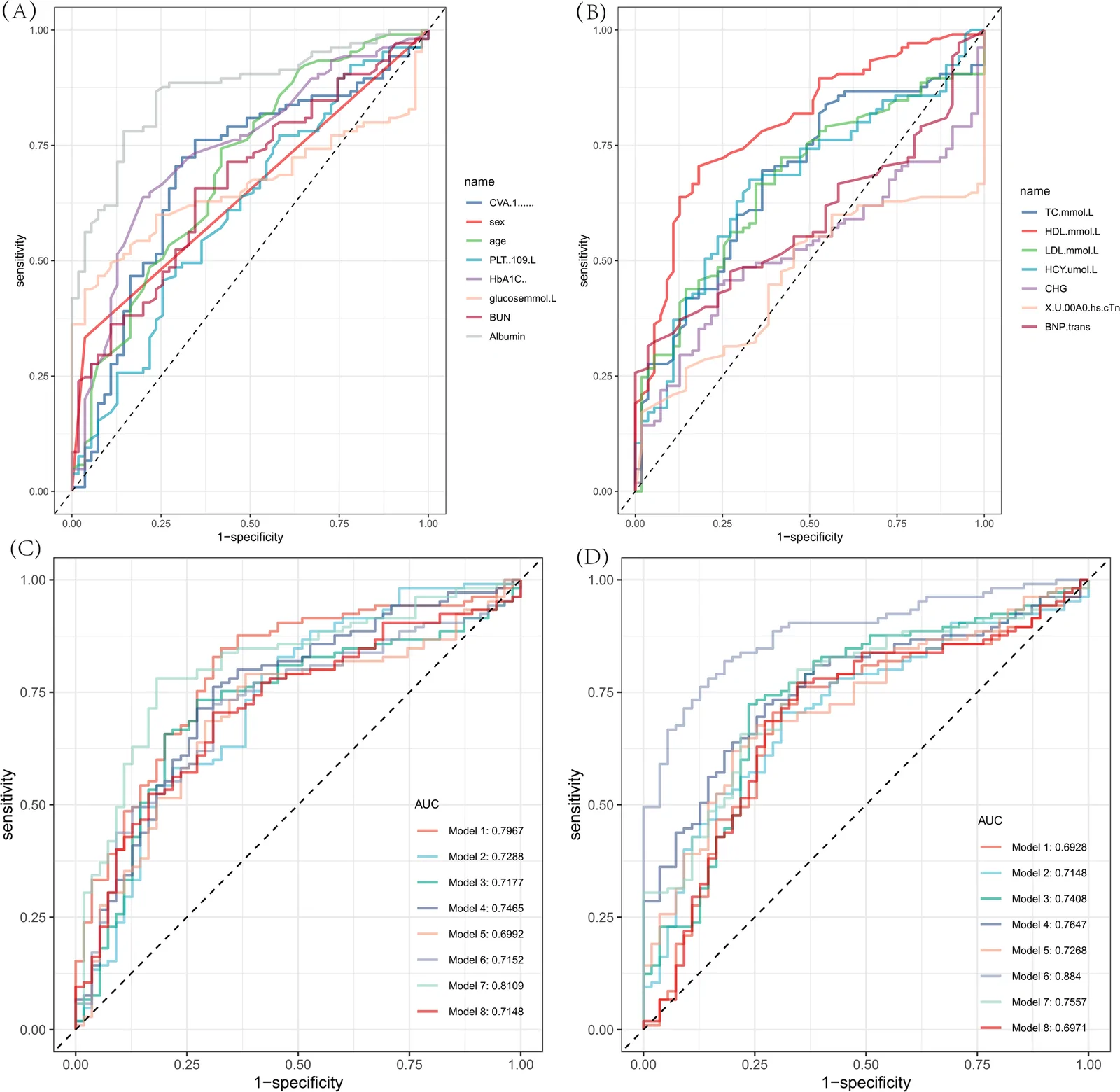
Receiver operating characteristic curves for predicting coronary heart disease. **(A)** Individual predictors: demographic, haematological, and metabolic markers. Highest AUCs: albumin 86.7% (orange), HbA1c 74.0% (pink), age 69.9% (turquoise), Caveolin-1 69.3% (red). **(B)** Individual predictors: lipid and cardiac/renal biomarkers. Highest AUC: HDL cholesterol 79.5% (pink). High-sensitivity cardiac troponin showed poor discrimination (46.6%, blue). **(C)** Caveolin-1 plus individual biomarkers. Model 1: Caveolin-1 alone (69.3%); Model 2: +LDL cholesterol (71.5%); Model 3: +homocysteine (74.1%); Model 4: Thigh-sensitivity cardiac troponin (76.5%); Model 5: +blood urea nitrogen (72.7%); Model 6: +albumin (88.4%, red); Model 7: +B-type natriuretic peptide (75.6%); Model 8: +haemoglobin (69.7%). **(D)** Caveolin-1 plus demographic and metabolic markers. Model 1: Caveolin-1 + sex (79.7%); Model 2: +age (72.9%); Model 3: +platelet count (71.8%); Model 4: +HbA1 c (74.7%); Model 5: +fasting glucose (69.9%); Model 6: +total cholesterol (71.5%); Model 7: +HDL cholesterol (81.1%, red); Model 8: +LDL cholesterol (71.5%). AUC, area under the curve (%); HbA1c, glycated haemoglobin; HDL, high-density lipoprotein; LDL, Iow-density lipoprotein.

**Figure 3:**
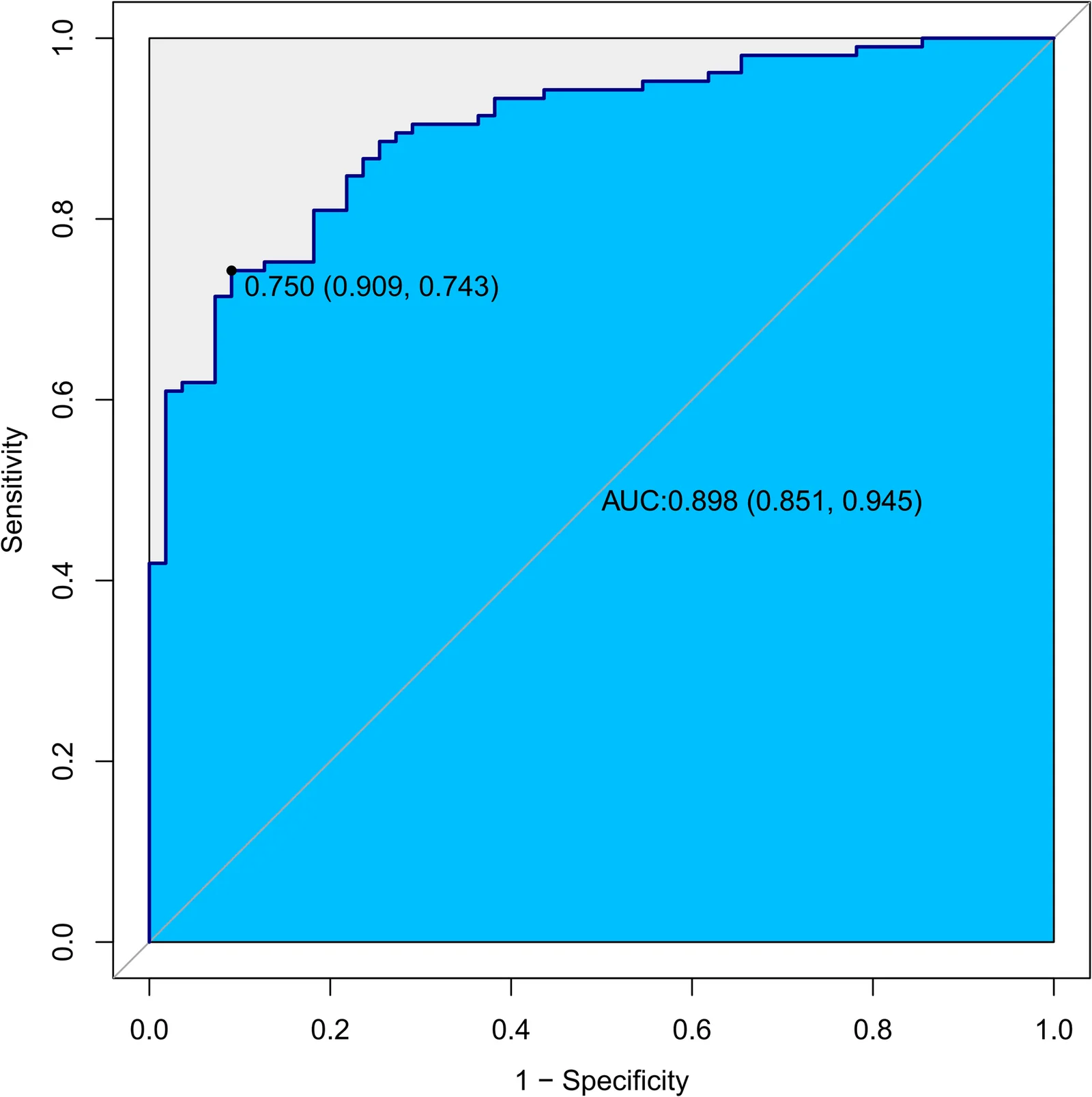
Receiver operating characteristic curve for the full multivariable model predicting coronary heart disease. Receiver operating characteristic curve for the full multivariable model (Model 3: Caveolin-1 + age + sex + total cholesterol + HDL cholesterol + B-type natriuretic peptide + blood urea nitrogen).

As visually summarized in Figure 2 and Table 4, albumin demonstrated the highest individual predictive accuracy (AUC 86.7%, 95% CI 81.2–92.3), followed by HDL-C (79.5%) and HbA1c (74.0%). Cav-1 alone showed modest discrimination (AUC 69.3%, 60.4–78.1). Combining Cav-1 with albumin significantly improved classification (AUC 88.4%, 83.3–93.5), while integration with HDL-C yielded an AUC of 81.1% (74.1–88.1). The full multivariable model (Figure 3), incorporating Cav-1, age, sex, lipids, and biomarkers, achieved optimal discrimination (AUC 89.8%, 5.1–94.5). Notably, combinations with hs-cTn (+7.2% vs. Cav-1 alone) and BNP (+6.3%) also enhanced predictive capacity, whereas routine hematologic indices (e.g., platelet count) provided marginal improvement. These findings highlight the synergistic diagnostic value of integrating Cav-1 with albumin or cardiometabolic biomarkers.

**Table 4:** Discriminative performance of caveolin-1 and clinical variables for predicting coronary heart disease.

**Variable**	**AUC, % (95% CI)**
Individual predictors	
Albumin, g/L	86.7 (81.2 to 92.3)
HDL cholesterol, mmol/L	79.5 (72.4 to 86.7)
HbA1c, %	74.0 (66.0 to 82.1)
Age, years	69.9 (61.2 to 78.6)
Caveolin-1	69.3 (60.4 to 78.1)
Total cholesterol, mmol/L	68.0 (59.5 to 76.5)
LDL cholesterol, mmol/L	67.1 (58.6 to 75.6)
Homocysteine, μmol/L	67.1 (58.6 to 75.7)
Blood urea nitrogen, mmol/L	67.0 (58.5 to 75.5)
Fasting glucose, mmol/L	66.3 (58.1 to 74.5)
Sex	64.8 (59.7 to 70.0)
Platelet count, ×10⁹/L	61.1 (51.9 to 70.4)
BNP	58.9 (50.2 to 67.6)
CHG	52.4 (43.5 to 61.3)
High-sensitivity cardiac troponin	46.6 (37.8 to 55.5)
Caveolin-1 plus individual covariates	
+ Albumin	88.4 (83.3 to 93.5)
+ HDL cholesterol	81.1 (74.1 to 88.1)
+ Sex	79.7 (72.4 to 87.0)
+ High-sensitivity cardiac troponin	76.5 (69.1 to 83.9)
+ BNP	75.6 (67.9 to 83.2)
+ HbA1c	74.7 (66.5 to 82.8)
+ Homocysteine	74.1 (65.7 to 82.4)
+ Age	72.9 (64.3 to 81.5)
+ Blood urea nitrogen	72.7 (64.7 to 80.7)
+ Platelet count	71.8 (63.3 to 80.3)
+ Total cholesterol	71.5 (63.3 to 79.8)
+ LDL cholesterol	71.5 (63.3 to 79.7)
+ Fasting glucose	69.9 (61.4 to 78.5)
+ CHG	69.7 (60.8 to 78.6)
Multivariable model	
Full modelᵃ	89.8 (85.1 to 94.5)

Full model includes Caveolin-1, age, sex, total cholesterol, H*D*L cholesterol, B-type natriuretic peptide, and blood urea nitrogen (corresponding to Model 3 in Table 3). Data represent area under the receiver operating characteristic curve (AUC) with 95% confidence intervals (CI) for predicting coronary heart disease. Individual predictors were ranked by AUC values in descending order within each category. The full multivariable model achieved the highest discriminative performance (AUC 89.8%), surpassing all individual predictors and two-variable combinations, indicating substantial synergistic value of combining Caveolin-1 with multiple clinical markers.

AUC, area under the curve; CI, confidence interval; HbA1c, glycated h*a*emoglobin; HDL, high-density lipoprotein; LDL, low-density lipoprotein.

To formally assess whether Cav-1 provided predictive information beyond albumin, we compared the reference model (age + albumin, AUC = 0.882) with an extended model additionally incorporating Cav-1 (AUC = 0.893). The results of reclassification and discrimination improvement analyses are presented in Supplementary Table S1.

The addition of Cav-1 significantly improved continuous NRI [0.755 (95% CI: 0.452–1.058), *p* < 0.001] and IDI [0.023 (95% CI: 0.0003–0.046), *P* = 0.047], indicating that Cav-1 enhanced both risk reclassification and overall discrimination beyond albumin alone. Categorical NRI did not reach statistical significance [0.092 (95% CI: −0.025–0.209), *p* = 0.124], likely reflecting its sensitivity to risk threshold selection. Although the AUC difference was not statistically significant (ΔAUC = 0.011, *p* = 0.175), this is anticipated given the high baseline discrimination (AUC = 0.882), which creates a ceiling effect limiting detectable AUC increments (11). These findings collectively support the independent incremental predictive value of Cav-1.

### Subgroup analysis: association of Cav-1 with CAD by subgroup

Subgroup analyses across key metabolic and demographic strata are presented in Table 5. The inverse association between Cav-1 and CAD was consistent among non-diabetic (OR = 0.77, *P* = 0.003), hypertensive (OR = 0.75, *P* = 0.018), and overweight/obese participants (BMI ≥ 24 kg/m^2^: OR = 0.73, *P* = 0.004). The association was attenuated and non-significant in the diabetic subgroup (OR = 1.07, *P* = 0.685), with a borderline interaction (*P* for interaction = 0.057). No significant interactions were identified for sex, BMI, or hypertension strata (all *P* for interaction >0.05).

**Table 5:** Association between CAV-1 level and risk of coronary heart disease across prespecified subgroups.

**Subgroup**	**Category**	**Total, n**	**Events, *n* (%)**	**Crude OR (95% CI)**	***P* value**	**Adjusted OR (95% CI)**	***P* value**	***P* for interaction**
Sex	Male	123	70 (56.9%)	0.77 (0.66–0.90)	0.001	0.80 (0.68–0.93)	0.005	0.379
	Female	37	35 (94.6%)	0.97 (0.58–1.63)	0.923	1.00 (0.62–1.62)	0.996	
BMI, kg/m²	<24	69	38 (55.1%)	0.89 (0.75–1.05)	0.154	0.92 (0.72–1.18)	0.512	0.174
	≥24	91	67 (73.6%)	0.73 (0.60–0.90)	0.003	0.73 (0.58–0.90)	0.004	
Diabetes mellitus	No	120	72 (60.0%)	0.80 (0.69–0.92)	0.003	0.77 (0.65–0.92)	0.003	0.057
	Yes	40	33 (82.5%)	1.05 (0.75–1.47)	0.765	1.07 (0.77–1.50)	0.685	
Hypertension	No	83	43 (51.8%)	0.93 (0.79–1.10)	0.388	0.92 (0.76–1.11)	0.390	0.183
	Yes	77	62 (80.5%)	0.72 (0.58–0.91)	0.005	0.75 (0.60–0.95)	0.018	

OR, odds ratio; CI, confidence interval; BMI, body mass index; CHD, coronary heart disease; kg/m², kilograms per square metre. Adjusted ORs were estimated after adjustment for age and sex, except for sex-stratified analyses, which were adjusted for age only. *P* values for interaction were derived from the corresponding adjusted models and are presented once for each subgroup variable.

## Discussion

This retrospective cohort study from Shanxi Provincial People's Hospital demonstrates three principal findings regarding serum Cav-1 and CAD in Chinese adults. First, Cav-1 levels exhibit an independent, inverse dose-response relationship with CAD risk, with each 1 ng/mL increase conferring an 18% risk reduction (adjusted OR 0.82, 95% CI 0.68–0.98, *P* = 0.031). Second, RCS analyses confirm a linear protective effect without threshold saturation (*P* for nonlinearity = 0.812), contrasting with the U-shaped curves often observed for metabolic biomarkers. Third, Cav-1 significantly enhances CAD discrimination when combined with albumin (AUC 88.4%, 95% CI 83.3–93.5) or HDL cholesterol (AUC 81.1%, 74.1–88.1), yielding improvements of 19.1% and 11.8% respectively vs. Cav-1 alone (AUC 69.3%). To our knowledge, this is the first study to quantify Cav-1's cardioprotective association in a Chinese cohort using multivariable-adjusted models, validate its linear dose-response via RCS with optimal knot placement (10th, 50th, and 95th percentiles), and systematically evaluate its incremental predictive utility alongside 15 routine clinical and biochemical biomarkers.

It is noteworthy that Cav-1 is not only a structural protein of plasma membrane caveolae but also a key regulator of multiple signaling pathways, including the mineralocorticoid receptor (MR). Recent reviews indicate that Cav-1 interacts with MR in cardiovascular tissues, and deficiency of Cav-1 can lead to overactivation of MR signaling, thereby promoting oxidative stress, inflammation, and fibrosis (12). Furthermore, Fernández-Hernando et al. demonstrated that endothelial Cav-1 deficiency accelerates atherosclerosis in ApoE^−/−^ mice (4), while Ramírez et al. found that Cav-1 inhibits LDL transcytosis independently of eNOS (6), Our data translate these mechanisms into human evidence: each unit elevation in circulating Cav-1 corresponded to 18% lower CAD odds after adjusting for traditional risk factors including lipid parameters (Model 3: OR 0.82, 95% CI 0.68–0.98). This lipid-independent protection suggests Cav-1 operates through pathways beyond conventional lipid metabolism.

Critically, restricted cubic spline analysis revealed a linear dose-response relationship (*P* for nonlinearity = 0.812), contrasting with prior genetic studies that identified Cav-1 variants without quantifying circulating protein levels (7). Protective associations emerged at Cav-1 levels as low as 5 ng/mL (adjusted OR 3.5, 95% CI 1.2–10.5) and intensified progressively through 12 ng/mL (OR 0.3, 95% CI 0.1–0.9), without saturation. This linearity distinguishes Cav-1 from HDL-C, where U-shaped mortality associations exist (13, 14), and implies that even modest Cav-1 elevations may confer cardiovascular benefit without requiring target thresholds. Quartile-based analyses corroborated these findings: participants in the highest Cav-1 quartile (≥8.72 ng/mL) experienced 80% CAD risk reduction vs. the lowest quartile (≤6.00 ng/mL) (Model 3: OR 0.20, 95% CI 0.05–0.84, *P* = 0.028; *P* for trend <0.01) with associations persisting after multivariable adjustment.

Our findings contrast with studies reporting elevated Cav-1 in metabolic syndrome and type 2 diabetes, where higher circulating Cav-1 associates with insulin resistance and cardiometabolic dysfunction (15). This paradox may reflect disease stage-specific biology: in early metabolic dysregulation, Cav-1 release from adipocytes represents a compensatory response to insulin resistance (16, 17), whereas in established CAD, low Cav-1 reflects endothelial dysfunction. Our cohort's lower diabetes prevalence (HbA1c: 6.3 ± 0.9% in CAD vs. 5.6 ± 0.7% in controls, *P* = 0.01) suggests circulating Cav-1 predominantly derives from endothelium, explaining the observed protective association.

Subgroup analyses further revealed that the inverse association between Cav-1 and CAD was attenuated in participants with diabetes mellitus (OR = 1.07,*P* = 0.685), with a borderline interaction (P_interaction = 0.057). This finding is biologically plausible. In the diabetic milieu, advanced glycation end-products and chronic oxidative stress impair Cav-1 expression and disrupt caveolar membrane integrity (18), potentially uncoupling Cav-1 from its cardioprotective eNOS-scaffolding and anti-inflammatory functions (19). Additionally, in diabetic states, Cav-1 is critically involved in insulin receptor signaling and may be functionally redirected toward metabolic compensatory roles rather than vascular protection (15), which could attenuate its discriminative value as a CAD biomarker. In contrast, the association remained robust among non-diabetic participants (OR = 0.77, *P* = 0.003), hypertensive participants (OR = 0.75, *P* = 0.018), and those with BMI ≥ 24 kg/m^2^ (OR = 0.73, *P* = 0.004), with no significant interactions detected in these strata (all *P* for interaction >0.05). These findings suggest that Cav-1 retains its cardioprotective signal across most metabolic subgroups, with diabetes status representing a potential effect modifier warranting further investigation in larger dedicated cohorts.

A recent study (20) reported a detrimental role of Cav-1 in chronic endothelial inflammation, which may appear discordant with our findings. However, these observations are not necessarily contradictory, as they reflect fundamentally different biological contexts. Tissue-expressed Cav-1 directly mediates pro-inflammatory intracellular signaling within the endothelium, whereas circulating serum Cav-1—as measured here—likely reflects systemic compensatory responses that do not mirror local pathological processes. Moreover, Cav-1's effects are inherently cell-type-, stage-, and context-dependent: its role may shift from pro-inflammatory in early endothelial injury to protective in established CAD, and differences in study design (experimental vs. observational) and measurement modality (tissue expression vs. serum levels) preclude direct comparison. Together, these findings underscore the pleiotropic nature of Cav-1 in cardiovascular biology and the need for mechanistic studies bridging experimental and clinical evidence.

Population-specific genetic factors may further modulate Cav-1's effects. CAV1 polymorphism rs3807992 interacts with dietary patterns to modify cardiometabolic risk. In overweight and obese women, A-allele carriers following healthy dietary patterns showed improved lipid profiles (higher HDL-C, lower TC/HDL and hs-CRP) compared to GG homozygotes (P for interaction = 0.03–0.04) (21). Asian populations' unique metabolic profiles—including higher carbohydrate intake, lower baseline HDL-C, and distinct apolipoprotein variants—could alter Cav-1-lipid interactions (22). Notably, our CAD cohort showed paradoxically lower LDL-C than controls (2.2 ± 0.9 vs. 2.6 ± 0.6 mmol/L, *P* = 0.008), which is most plausibly explained by the markedly higher prevalence of statin use in the CAD group (86.3% vs. 25.5%, *P* < 0.001). Beyond lipid lowering, statins suppress Cav-1 expression and promote eNOS dissociation from caveolae via PI3K/Akt-dependent mechanisms, thereby disinhibiting eNOS activation (23, 24). We therefore cannot exclude that a portion of the lower Cav-1 observed in CAD patients reflects statin-mediated suppression rather than intrinsic endothelial pathology alone. This consideration is addressed further in the Limitations. These observations collectively underscore the need for longitudinal studies capturing pre-diagnostic, statin-naïve Cav-1 levels to establish the true temporal and causal relationship between circulating Cav-1 and CAD.

The biological plausibility of Cav-1's protective role is supported by converging mechanistic pathways. First, Cav-1 scaffolds eNOS at caveolar membranes, facilitating phosphorylation and NO production upon agonist stimulation (5). Cav-1-null mice exhibit eNOS mislocalization, causing excessive basal NO but blunted agonist-stimulated responses—a dysregulated phenotype predisposing to endothelial dysfunction. Second, Cav-1 interacts with SR-BI in HDL-mediated cholesterol efflux, though the relationship remains debated (25, 26). Our observation that Cav-1 synergizes with HDL-C in CAD prediction (combined AUC 81.1% vs. Cav-1 alone 69.3%, +11.8%) suggests cooperative pathway operation. Third, Cav-1 suppresses pro-inflammatory NF-κB signaling, attenuating vascular inflammation (27).

Beyond endothelial effects, Cav-1's suppression of NF-κB signaling may also modulate macrophage-driven atherogenesis. NF-κB activation is known to impair efferocytosis by downregulating phagocytic receptors and disrupting mitochondrial homeostasis in macrophages. Macrophage efferocytosis—the clearance of apoptotic cells within atherosclerotic plaques—is a key determinant of plaque stability regulated by epigenetic and transcriptional mechanisms (28), and impaired efferocytosis promotes necrotic core expansion and plaque vulnerability. Concurrently, the survival of tissue-resident macrophages in the chronic inflammatory environment of atherosclerotic lesions depends on mitochondrial integrity, a process modulated by factors such as SerpinB2 (29). Thus, Cav-1-mediated NF-κB suppression may indirectly preserve both efferocytic capacity and macrophage metabolic fitness, such that higher circulating Cav-1 levels reflect a systemic anti-inflammatory state that contributes to plaque stabilization. This macrophage-Cav-1 interaction represents an important avenue for future investigation, particularly in studies with plaque-level histological data.

The synergistic interaction between Cav-1 and albumin (combined AUC 88.4%, approaching the full model's 89.8%) merits emphasis. Albumin stabilises the endothelial glycocalyx, essential for mechanotransduction and NO production (30, 31). and enhances shear-stress-induced, NO-mediated vasodilation by binding glycocalyx components (32). In our CAD cohort, albumin was significantly lower (39.3 ± 3.5 vs. 43.8 ± 2.7 g/L, *P* < 0.001), likely reflecting chronic inflammation (elevated homocysteine: 17.8 ± 10.1 vs. 13.2 ± 5.3 μmol/L, *P* = 0.014). Although both biomarkers may share upstream pathways, they reflect distinct aspects of vascular pathology: Cav-1 regulates transcytosis, NO signalling and permeability through caveolae, capturing early endothelial dysfunction; albumin, as a major glycocalyx constituent shed upon degradation, reflects downstream structural glycocalyx loss. Supporting this mechanistic complementarity, simultaneous adjustment confirmed independent inverse associations with CAD (Cav-1: adjusted OR 0.77, 95% CI 0.64–0.93, *P* = 0.006; albumin: adjusted OR 0.54, 95% CI 0.43–0.67, *P* < 0.001)(Supplementary Table S3), with a low Spearman correlation (*r* = 0.19, *p* = 0.014) and variance inflation factors of 1.215 and 1.209(Supplementary Tables S2, S4), excluding multicollinearity. Incremental analysis showed a modest AUC gain (ΔAUC = 0.011, *P* = 0.175) but significant improvements in continuous NRI (0.755, *p* < 0.001) and IDI (0.023, *P* = 0.047), indicating that Cav-1 provides meaningful reclassification and discrimination beyond albumin alone (Supplementary Table S1). This likely reflects a ceiling effect of the high-performing baseline model rather than absence of true incremental value, as NRI and IDI are more sensitive than ΔAUC for detecting novel biomarker contributions in such settings (10, 11). Thus, Cav-1 complements albumin by capturing distinct pathophysiological information related to endothelial dysfunction, lipid-raft signalling dysregulation and atherogenic processes not fully reflected by albumin. Combined low Cav-1 and albumin may identify a “high-risk endothelial phenotype” characterised by impaired eNOS regulation, glycocalyx degradation and heightened inflammation—features potentially amenable to targeted therapies.

From a translational perspective, Cav-1 adds meaningful discriminative value beyond established biomarkers. Albumin alone achieved the highest individual AUC (86.7%, 95% CI 81.2–92.3), followed by HDL-C (79.5%) and HbA1c (74.0%), whereas Cav-1 alone showed modest performance (69.3%, 60.4–78.1). However, combining Cav-1 with albumin elevated AUC to 88.4% (83.3–93.5), nearly equivalent to the full multivariable model incorporating 15 variables (89.8%, 85.1–94.5). This suggests a parsimonious two-biomarker panel could efficiently stratify CAD risk in resource-limited settings, though optimal clinical thresholds require validation in independent cohorts. Integration into existing risk algorithms offers clinical utility. The China-PAR score, validated for 10-year atherosclerotic cardiovascular disease prediction in Chinese populations (33), incorporates traditional risk factors but lacks proteomic biomarkers. Adding Cav-1 and albumin as continuous variables could refine intermediate-risk classification (10-year risk 10%–20%), where treatment decisions are most uncertain. Alternatively, Cav-1 measurement could be reserved for patients with discordant traditional risk factors (e.g., normal LDL-C but positive family history), where additional biomarkers provide actionable information.

The main strengths of this study include rigorous coronary angiography-based CAD definition, systematic covariate adjustment (VIF < 2.0), and nonlinear RCS modeling. Several limitations should be acknowledged. First, the single-center design, unmeasured confounders (e.g., physical activity, diet, and medications), and cross-sectional nature limit generalizability, causal inference, and adjustment. Second, coronary angiography relied on descriptive stenosis estimates rather than standardized quantification, preventing calculation of Gensini or SYNTAX scores. Third, the control group from a health examination center may introduce a healthy-worker bias that could overestimate Cav-1's discriminative performance. Fourth, statin use was markedly higher in the CAD group (86.3% vs. 25.5%), and its near-collinearity with CAD status precluded full adjustment; since statins may lower Cav-1 via eNOS-dependent mechanisms, the observed inverse association could be partially confounded. Fifth, a notable sex imbalance existed (male proportion: 66.7% in CAD vs. 96.4% in controls), likely reflecting referral patterns; although sex adjustment and male-stratified analyses yielded consistent findings, the small female sample (*n* = 37) precludes definitive conclusions for women. Sixth, we acknowledge that we did not independently conduct additional dilution linearity or matrix effect assessments for the Cav-1 ELISA kit in our own laboratory. However, the kit is a commercially available product with comprehensive validation data provided by the manufacturer, including spike recovery (85%–102%), intra-assay CV (<5.16%), and inter-assay CV (<4.88%), all of which meet or exceed internationally accepted standards. All samples were processed under identical conditions strictly following the manufacturer's protocol, which effectively minimized technical variability. Future studies with population-based controls, quantitative coronary angiography, prospective designs, and alternative assay validation protocols are needed to validate our results and provide more robust dose-response evidence.

## Conclusions

In conclusion, this study establishes circulating Cav-1 as an independent, dose-dependent protective factor against CAD in Chinese adults, exhibiting a linear inverse relationship without threshold saturation across physiological concentrations. Its robust performance when combined with albumin (AUC 88.4%) underscores potential dual utility as both a biomarker for refined risk stratification and a mechanistic indicator of integrated endothelial-metabolic dysfunction. These findings challenge the uniform “harmful caveolin” paradigm observed in metabolic syndrome cohorts, highlighting context-dependent roles influenced by disease stage (early metabolic dysfunction vs. established atherosclerosis) and tissue origin (adipose vs. endothelial) that merit population-specific investigation. If validated prospectively in multicenter cohorts and supported by mechanistic biomarker studies, Cav-1 measurement—particularly in combination with routine albumin assessment—could enhance identification of high-risk individuals who would benefit most from intensive preventive interventions, ultimately advancing personalized approaches to CAD prevention in Chinese and broader Asian populations where unique genetic and dietary factors may modulate caveolar biology.

## Data Availability

The raw data supporting the conclusions of this article will be made available by the authors, without undue reservation.
