# Supplementary material for: Caveolin-1 levels associated with coronary artery disease in Chinese participants: a retrospective cohort study
Source: Front Cardiovasc Med. 2026 Aug 7;13:1790930. doi: 10.3389/fcvm.2026.1790930 (PMC13493251; doi:10.3389/fcvm.2026.1790930)
Supplement: Supplementary file 1 [file Supplementaryfile1.docx]

**Supplementary Table 1. Incremental predictive value of Caveolin‑1 beyond albumin: comparison of reference and extended models.**

| **Metric** | **Reference model (Age + Albumin)** | **Extended model (Age + Albumin + Cav-1)** | **Improvement [95% CI]** | **P value** |
| --- | --- | --- | --- | --- |
| AUC | 0.882 | 0.893 | ΔAUC = 0.011 | 0.175 |
| NRI (Categorical) | — | — | 0.0918 [−0.0253, 0.2089] | 0.124 |
| NRI (Continuous) | — | — | 0.755 [0.4517, 1.0582] | <0.001 |
| IDI | — | — | 0.023 [0.0003, 0.0457] | 0.047 |

*Reference model included age and serum albumin. Extended model additionally included caveolin-1 (CAV-1). AUC, area under the receiver operating characteristic curve; NRI, net reclassification improvement; IDI, integrated discrimination improvement; CI, confidence interval. Improvement estimates were calculated as the difference between the extended and reference models. Two-sided P values are reported.*

**Supplementary Table 2. Correlation between Caveolin‑1 level and serum albumin concentration**

| **Variable** | **Correlation coefficient, r** | **95% CI** | **P value** |
| --- | --- | --- | --- |
| **Cav-1 level** | 1.000 | — | — |
| **Serum albumin, g/L** | 0.193 | Not reported | 0.014 |

*Correlation coefficients were estimated using Pearson correlation analysis. The correlation between Cav-1 level and serum albumin concentration was weak but statistically significant (r=0.193; P=0.014). CI, confidence interval.*

**Supplementary Table 3. Multivariable logistic regression analysis for the independent**

**associations of serum albumin and Caveolin‑1 with CAD.**

| **Variable** | **OR (95%CI)** | **P value** |
| --- | --- | --- |
| Albumin^1^ | 0.54 (0.43~0.67) | <0.001 |
| Cav-1^2^ | 0.77 (0.64~0.93) | 0.006 |

*Two separate logistic regression models were constructed. In Model 1, albumin was entered as the primary exposure variable, with Cav‑1, age, and sex included as covariates.*

*In Model 2, Cav‑1 was entered as the primary exposure variable, with albumin, age, and sex included as covariates. Odds ratios (OR) and 95% confidence intervals (CI) are presented.*

**Supplementary Table 4. Multicollinearity assessment and coefficient stability between Caveolin‑1 and albumin after mutual adjustment.**

| **Term1 (Crude)** | **Coeff1** | **Change % 1** | **Term2 (Full)** | **Coeff2** | **Change % 2** | **VIF** |
| --- | --- | --- | --- | --- | --- | --- |
| Cav-1 | −0.20 | Ref. | Full | −0.25 | Ref. | 1.215 |
| age | −0.16 | −18.1% | age | −0.27 | 8.4% | 1.031 |
| sex | −0.24 | 19.1% | sex | −0.16 | −35.4% | 1.281 |
| Albumin | −0.19 | −3.3% | Albumin | −0.20 | −19.4% | 1.209 |

*Variance inflation factors (VIF) were calculated from the mutually adjusted model (age + sex + Cav‑1 + albumin), with values well below the conventional threshold of 5, confirming the absence of multicollinearity between the two biomarkers*
